# Contribution of Human Retroviruses to Disease Development—A Focus on the HIV– and HERV–Cancer Relationships and Treatment Strategies

**DOI:** 10.3390/v12080852

**Published:** 2020-08-04

**Authors:** Ching-Hsuan Liu, Nicole Grandi, Lalitha Palanivelu, Enzo Tramontano, Liang-Tzung Lin

**Affiliations:** 1 Graduate Institute of Medical Sciences, College of Medicine, Taipei Medical University, Taipei 11031, Taiwan; d119107007@tmu.edu.tw; 2 Department of Microbiology & Immunology, Dalhousie University, Halifax, NS B3H 4R2, Canada; 3 Department of Life and Environmental Sciences, University of Cagliari, Monserrato, 09042 Cagliari, Italy; nicole.grandi2@gmail.com (N.G.); tramon@unica.it (E.T.); 4 International Master Program in Medicine, College of Medicine, Taipei Medical University, Taipei 11031, Taiwan; lalitaniyaa62@gmail.com; 5 Department of Microbiology and Immunology, School of Medicine, College of Medicine, Taipei Medical University, Taipei 11031, Taiwan

**Keywords:** human endogenous retroviruses, human immunodeficiency virus, carcinogenesis, cancer treatment, antiviral strategies, immunotherapy

## Abstract

Animal retroviruses are known for their transforming potential, and this is also true for the ones hosted by humans, which have gathered expanding attention as one of the potent causative agents in various disease, including specific cancer types. For instance, Human T Lymphotropic virus (HTLV) is a well-studied class of oncoviruses causing T cell leukemia, while human immunodeficiency virus (HIV) leads to acquired immunodeficiency syndrome (AIDS), which is linked to a series of defining cancers including Kaposi sarcoma, certain types of non-Hodgkin lymphoma, and cervical cancer. Of note, in addition to these “modern” exogenous retroviruses, our genome harbors a staggering number of human endogenous retroviruses (HERVs). HERVs are the genetic remnants of ancient retroviral germline infection of human ancestors and are typically silenced in normal tissues due to inactivating mutations and sequence loss. While some HERV elements have been appropriated and contribute to human physiological functions, others can be reactivated through epigenetic dysregulations to express retroviral elements and promote carcinogenesis. Conversely, HERV replication intermediates or protein products can also serve as intrinsic pathogen-associated molecular patterns that cause the immune system to interpret it as an exogenous infection, thereby stimulating immune responses against tumors. As such, HERVs have also been targeted as a potential internal strategy to sensitize tumor cells for promising immunotherapies. In this review, we discuss the dynamic role of human retroviruses in cancer development, focusing on HIV and HERVs contribution. We also describe potential treatment strategies, including immunotherapeutic targeting of HERVs, inhibiting DNA methylation to expose HERV signatures, and the use of antiretroviral drugs against HIV and HERVs, which can be employed as prospective anti-cancer modalities.

## 1. Introduction

Retroviruses are a large group of viruses that cause a wide range of diseases, including cancers. Accordingly, the well-described transforming nature of animal retroviruses led to their original definition as “RNA tumor viruses” [1]. Currently, there are two exogenous retroviruses affecting human health: human immunodeficiency virus (HIV) and human T lymphotropic virus (HTLV). While HTLV is a “classical” oncovirus, causing T cell leukemia as its main etiological manifestation, HIV infection is responsible for acquired immunodeficiency syndrome (AIDS), which is accompanied by a number of comorbidities, including increased incidence of different types of cancers [2].

Of note, in addition to the above exogenous retroviruses, the DNA of all vertebrate contains endogenous retroviruses (ERVs), which are ancient traces of past infections found as viral footprints in the genome of the various species. The ones found in humans, the human endogenous retroviruses (HERVs), represent as approximately 8% of the human genome and have been recently classified into 39 main groups [3]. These viral footprints are virus-associated sequences that closely resemble present-day retroviral (e.g., HIV) elements, including the 5’ and 3’ long terminal repeats (LTR), and the coding genes *gag*, *pro-pol*, and *env* [4]. However, their long-time persistence in the host genome has led to the accumulation of mutations as well as insertions and deletions, which have generally affected their capacity to produce infectious virions [5].

Viruses of the *Retroviridae* family typically contain two copies of positive-sense single-stranded RNA (ssRNA) genome at their core and are surrounded by host-derived lipid membrane inserted with the *env* gene-encoded glycoproteins (e.g., gp160 in the case of HIV, which is processed to yield the surface gp120 and the transmembrane gp41 [6]). The Env glycoproteins mediate the entry steps of HIV into the targeted host cells, whereas the viral enzymatic activities (reverse transcriptase (RT), RNase H, integrase (IN), and protease (PR)) encoded by the *pro-pol* gene are crucial to viral replication [6]. Retroviruses are characterized by their ability to perform reverse transcription of their ssRNA genome and integrating them into the host chromosomal DNA [7]. As such, ERVs are considered molecular remnants of ancient exogenous retroviruses resulted from their germ line infection and integration in vertebrate ancestors approximately over 100 million years ago [8]. 

In this review, we have included in the first part the HIV–cancer relationship and the associated use of antiretroviral therapy (ART) to demonstrate the potential applications of ART as anti-cancer strategies relevant to retroviruses due the solid evidence in this field, whereas the second part focuses on the new implication of HERV and the development of novel anti-cancer strategies for HERV-associated cancer scenarios. Although HTLV is also an onco-retrovirus, evidence for treating HTLV-associated T cell leukemia with antiretroviral therapy remains limited. Currently, only the combination of interferon (IFN)-α and zidovudine showed some benefits in patients with T cell leukemia, and the underlying mechanism and the antiviral contribution of zidovudine require further investigation (as reviewed by Marino-Merlo et al. [9] and Futsch et al. [10]). Therefore, the following discussion will be focused on HIV and HERV.

## 2. HIV-Associated Cancers: Classification and Epidemiology

When the first clusters of AIDS epidemics were observed, at the beginning of the 1980s, the manifestations associated with immunodeficiency included Kaposi’s sarcoma, aggressive B cell lymphomas, and invasive cervical cancer. These malignancies were commonly found in AIDS patients and thus became the “AIDS-defining” cancers when observed in HIV-infected individuals [11]. In addition to AIDS-defining cancers, a number of other malignancies are known to be associated to HIV infection, even if non-AIDS-defining, showing an increased incidence among HIV patients. These HIV-associated cancers include lung cancer, hepatocellular carcinoma, Hodgkin’s lymphoma, oropharyngeal cancers, and anal and genital cancers [2]. The overall augmented cancer risk in HIV-infected individuals is driven by multiple factors. In most of these conditions, HIV does not have a direct transforming role, but the effects of its infection, especially on the host immune system, create a dysregulated environment triggering cancer development by both immunosuppression, which can aid in the immune evasion of oncoviruses and cancer cells, and chronic inflammation, which promotes cellular proliferation hence tumorigenesis [12,13]. Behavioral aspects of the HIV-infected individuals, such as the increased rate of co-exposure to oncoviruses in people with multiple sexual contacts or drug users, also contribute to the increased cancer risk [14].

Notably, the development of effective antiretroviral drugs (ARVs) has led to a significant reduction of AIDS-defining cancers (discussed below). In turn, the concomitant improvement of treated patients’ survival has doubled the number of people living with AIDS, and a large proportion of them is currently in the age range that is associated with augmented cancer risk [15]. As a consequence of an aging HIV-infected population, the occurrence of both HIV-associated (non-AIDS-defining) cancers and common incidental cancers has increased, becoming the main cause of death in developed countries [2]. It is noteworthy that adolescents and young adults living with AIDS also have an increased risk of developing both AIDS-defining and non-AIDS-defining cancers compared to HIV-negative individuals of the same age, and their relatively low adherence to ARVs could aggravate the occurrence of malignancies [16].

## 3. AIDS-Defining HIV-Associated Cancers

The first cancer historically connected to AIDS’ presence was Kaposi’s sarcoma, characterized by lesions on the skin and other organs, including oral mucosa, gastrointestinal tract, lymph nodes, lungs, and bones [2]. Kaposi’s sarcoma is caused by human herpesvirus 8, also known as Kaposi’s sarcoma-associated herpesvirus (KSHV). KSHV does not require HIV infection to develop Kaposi’s sarcoma, but the immune dysregulation induced by HIV affects the immunologic control of KSHV and other oncoviruses found in HIV patients, sustaining carcinogenesis. Accordingly, the risk of Kaposi’s sarcoma in HIV patients is inversely related to their CD4+ T cell count, although a few patients with relatively high CD4+ T cell count still had persistent Kaposi’s sarcoma [17]. The second group of AIDS-defining cancer is represented by aggressive B cell non-Hodgkin’s lymphomas [2]. Of which, primary central nervous system lymphoma and plasmablastic lymphoma often consist of cells infected by Epstein Barr virus (EBV), while Burkitt’s lymphoma and diffuse large B cell lymphoma are mainly negative for EBV [2]. Although the overall incidence of non-Hodgkin lymphomas in HIV patients showed a pronounced decrease with the advent of effective antiretroviral treatments, it still remains 10-fold higher as compared to uninfected population [18,19]. The third and last AIDS-defining malignancy is invasive cervical cancer, whose risk is increased in HIV-infected women, being associated with age but not with CD4+ T cell count [20]. Overall, almost all cervical cancers arise from human papilloma virus (HPV) oncogenesis, and the concomitant presence of HIV may augment its incidence by affecting the host immune response and thus preventing the clearance of HPV infection [13].

## 4. Other HIV-Associated Cancers

Besides the above AIDS-defining cancers, HIV infection is associated with increased incidence of a number of other malignancies. The most frequent HIV-associated cancers include lung cancer, hepatocellular carcinoma, Hodgkin’s lymphoma, oropharyngeal cancers, as well as genital and anal cancers [2]. In the majority of cases, oncogenesis is mediated by the co-infection with an oncovirus, in which HIV-induced immune dysregulation facilitates its immune evasion and tumorigenesis.

A great proportion of HIV-associated cancers affecting the oropharynx, genitals and anus are linked to HPV infection as well [2,21]. In fact, as mentioned for cervical cancer, HIV-infected individuals present immunosuppression that is linked to a higher risk to establish a chronic HPV infection and develop pre-malignant high-grade intraepithelial lesions [13,22]. As for hepatocellular carcinoma, its etiological agent hepatitis virus B and C (HBV and HCV, respectively) are frequently transmitted with the same route of HIV, and thus often co-infect with HIV. This increases the incidence of hepatocellular carcinoma in HIV patients, with similar cancer progression in non-HIV-infected individuals with chronic viral hepatitis [13]. Concerning non-AIDS defining lymphomas associated to HIV infection, they are also often caused by the concomitant infection with transforming viruses, such as EBV and KSHV. Pharmacological therapy against HIV has not been able to reduce the incidence of Hodgkin lymphoma in infected individuals, but recent findings suggest that prolonged ART seems to reduce the associated risk [23]. Overall, besides a higher incidence of lymphomas, individuals infected with HIV tend to show extranodal and central nervous system involvement [24] and elevated cancer-specific mortality [25] as compared to the general population. Lung cancer is another malignancy reported to have higher incidence in HIV-infected individuals than the uninfected population [26]. There is a three-times increased risk in HIV-infected individuals, which depends in part on a high prevalence of smokers [27], but also on HIV-related factors such as elevated susceptibility to pneumonia and chronic obstructive pulmonary disease, which contribute to a higher risk of lung malignancies [26,28]. The associated mortality, however, seems not to be linked to either HIV load or CD4+ T cell count [13,29]. In addition to the above HIV-associated tumors, the presence of the infection augments the risk of incidental cancers as well, including colon, breast, prostate, and squamous-cell skin cancers [2].

## 5. Immunotherapeutic Strategies Targeting HIV for Cancer Treatment

Given the clear association between HIV infection and the increased incidence of human malignancies, a number of prevention and therapeutic strategies have been proposed. First of all, given that most HIV-associated tumors depend on co-infection with known oncoviruses, a significant cancer prevention clue is represented by the vaccinations available against the oncoviruses, such as HPV and HBV for cervical and hepatocellular cancers, respectively. Similarly, screening procedures are crucial for cancer prevention and early diagnosis, not only in HIV-infected patients but also in the general population. Another important action is the early diagnosis of HIV infection, allowing for the prompt initiation of effective ART before the drastic reduction of CD4+ T cell count. In fact, growing evidence suggests that early and uninterrupted ART can reduce the risk of cancer associated with HIV infection, restoring CD4+ T cell count and limiting oncoviruses’ immune evasion [15]. Accordingly, the risk of cancer was decreased by approximately two-thirds in HIV patients who started ART at a CD4+ T cell count higher than 500 cells/mm^3^ [13]. ART is an effective immunotherapy for AIDS-defining cancers, and immunotherapeutic strategies based on monoclonal antibodies, IFN, cytokines and immunomodulatory drugs have also shown efficacy in HIV-associated malignancies [30]. Finally, the prevention of behavioral factors that increase HIV-associated cancer incidence, such as smoking for lung cancer or unprotected sex for sexually transmitted oncoviruses, can play an important role in reducing the incidence of these malignancies in HIV-infected individuals.

## 6. The Physiological Implications of HERV Elements

As mentioned above, ERVs are viral footprints that are found in the host genomes. Over the time of evolution, integrated ERVs can be either lost or fixed (when the frequency of inserted ERV becomes 100% in the population), if the elements are beneficial for the host, as a result of natural selection and random mutations [8]. Owing to their ability to integrate into the host genome, ERV sequences have important implication in host phenotypic effects that can have consequences in the context of physiological functions and diseases [31]. For example, the syncytins in humans, namely syncytin-1 encoded by a HERV-W provirus *env* and syncytin-2 encoded by a HERV-FRD provirus *env*, are thought to have been acquired by the primates many millions ago (~25 million years ago for syncytin-1 and >40 million years ago for syncytin-2) [32,33] and are essential for placental morphogenesis. It has also been proposed that the syncytins can have immunomodulatory functions, including immunosuppression [34] and the inhibition of Th1 cytokine production [35], possibly through the putative immunosuppressive domain in their transmembrane subunit. The overall effect is thought to contribute to the physiological maternal tolerance for the fetus during pregnancy [34,35,36]. Owing to their critical physiological roles in the placenta, this domestication and appropriation of the HERV Envs exerts a positive selection for gene fixation or retention along the primate evolution, observing high degree of conservation and very limited human polymorphisms [36,37].

In addition, HERVs play a role in the regulation of human gene expression in several aspects. HERV elements serve as crucial regulatory factors for the pluripotency in embryonic stem cells [38] and are expressed at varied levels in normal human tissues [1,39] to facilitate tissue-specific gene expression. Furthermore, it is recently revealed that HERV-derived molecules modulate the innate immunity [4], which can elicit both positive antiviral protection against exogenous viruses and negative autoimmune and inflammatory disease-inducing effects (further discussed below). These observations highlight the importance of HERV as a subject of study to unravel the genetic evolution of various species, and more intriguingly, for deciphering the pathophysiological development of illnesses and even be potentially explored as therapeutic targets for the treatment of many present day diseases.

## 7. HERV Activation and the Paradigm of HERV in Cancers

In most tissues, HERVs are transcriptionally silenced by CpG methylation catalyzed by DNA methylase-1 [40]. The aberrant expression of HERV has been found in a number of human diseases, such as cancers [41], autoimmune diseases [42], and neurological diseases [43], possibly suggesting their role in pathogenesis. Current literature suggests that HERV can be activated by a plethora of microenvironmental stimuli such as hormones, cytokines, epigenetic modifications or exogenous microorganisms [44]. Interestingly, recent studies suggest that several viral infections, such as retroviruses (HIV, HTLV-1), herpesviruses (EBV, herpes simplex virus type 1 (HSV-1), KSHV), HBV, and influenza virus, can transactivate HERV [45]. These findings might provide clues to the pathogenicity of certain viruses, including those that lead to long-term disease development such as autoimmune diseases and cancers. 

In the case of cancer, a considerable level of early investigation has been focused on the oncogenic properties of retroviruses. Many studies attempted to illustrate the role of HERV in cancer [46], as HERV elements and HERV-encoded proteins were found in a variety of cancers including melanoma, breast cancer, colorectal cancer, hepatobiliary cancers, prostate cancer, ovarian cancer, and germ cell tumors [44,47,48]. As such, HERV elements have been proposed to be potential biomarkers for cancers and could have an implication for therapeutic intervention [48]. Although the average expression of HERV appears quite homogenous among different types of cancers [49], it is noteworthy that in some studies the overexpression of HERV was not found. For instance, in the analysis performed by Rooney et al., little overexpression of the “tumor-specific” HERVs (ERVH−5, ERVH48−1, and ERVE−4 in the study) was found in glioma and thyroid cancer [50]. In another study by Bergallo et al., while HERV-K *pol* was overexpressed in pediatric acute lymphoblastic leukemia (ALL) patients, the expression level was not significantly increased in those with acute myeloid leukemia (AML) [51]. In contrast, Januszkiewicz-Lewandowska et al. demonstrated that HERV-K *env* was overexpressed in AML but not ALL [52]. In both studies, HERV-W *pol* and *env* were not found to be associated with either AML or ALL [51,52]. These examples suggest that HERV elements may not be overexpressed in all cancer types, and that different groups and different parts of HERV may be activated in specific malignancies.

In addition, it remains controversial whether the presence of HERV has a direct causative role or if it is simply a bystander effect of epigenetic changes, such as the hypomethylation of DNA and chromatin remodeling in the cancer cells, which could expose previously silent HERV LTRs [53]. In contrast, the continued investigation has also revealed that HERVs may contribute to host defense responses against cancer [54], implicating their presence as participants rather than mere bystanders. As a result, the precise role of HERV in cancer development remains to be fully clarified, but several observations described below shed light to the evolving paradigm of HERV-cancer association.

## 8. HERV Contribution to Cancer at the Genome Level

Potentially oncogenic properties of HERV at the genome level include non-allelic recombination of HERV sequences, which results in chromosomal rearrangements, and HERV LTR as an alternative promotor to cellular gene expression that contributes to tumorigenesis [54]. Such effects were seen in prostate cancer [55], melanoma, Hodgkin’s lymphoma, diffuse large B cell lymphoma, etc. [53], where abnormal gene expression occurred due to HERV-induced translocation and/or LTR regulation. In addition, recent studies demonstrate that HERV-K(HML2) and HERV-H are concurrently expressed with pluripotent transcriptional factors NANOG, OCT-4, and SOX-2 in mesenchymal stem cells [56], while HERV-W has been detected in cancer-stem like colorectal cancer cells [57]. It has also been shown in melanoma cells that HERV-K(HML2) activation promotes phenotype-switching to obtain cancer stem-like features [58]. It would be interesting to explore whether HERV contributes to the stemness or pluripotency in cancer stem cells in a similar way as in embryonic stem cells, i.e., through LTR and non-coding RNA (ncRNA) regulation, due to the common regulatory factors they share [44].

## 9. HERV Contribution to Cancer at the Protein Level

At the protein level, some Env proteins promote the tumorigenesis due to their ability to trigger cell-to-cell fusion, immunosuppression, and cell signaling pathways that are crucial for cancer development [54]. In the case of breast cancer, HERV-K Env protein plays an important role in tumorigenesis and metastasis through activating the Ras-ERK pathway [59]. Anti-HERV-K Env antibodies and Env-directed chimeric antigen receptor (CAR) T cells exhibited significant tumor-specific cytotoxicity and prevented metastasis in mouse xenograft models [60,61]. In the case of melanoma, a HERV-K(HML6) Env spliced variant, HERV-K-Mel, was found to be targeted by cytotoxic T cells in melanoma patients [62]. HERV-K Env-specific CAR T cells similarly reduced tumor burden and metastasis in mouse xenograft models [63]. Research in pancreatic cancer also demonstrated that HERV-K(HML2) Env regulates the Ras-ERK pathway, and knockdown of Env in vitro and in vivo reduced the growth of pancreatic cancer and metastasis [64]. Finally, the *env*-encoded H17 protein of HERV-H was found to induce epithelial-to-mesenchymal transition (EMT) in tumor cells and recruit immunoregulatory suppressor cells, suggesting its contributory role in tumor metastasis and immune escape [65]. These findings support the role of HERV Env as a tumor-promoting antigen in various types of cancers. In addition, the two spliced variants encoded by HERV-K(HML2) *env* gene, Rec and Np9 accessory proteins, may also play a role in transformation by binding to functional cellular proteins [54], such as the transcriptional repressor of the c-MYC proto-oncogene [66], proteins involved in androgen receptor repression [67,68], and the ligand of Numb protein X (LNX) [69] involved in Notch signaling. Of note, the presence of a coding sequence for Rec putative protein has been recently reported also for HERV-K HML10 and HML6 groups [70,71].

## 10. HERV-Induced Immune Responses—Implication for Cancer Management

Since HERV elements are transcriptionally controlled in normal tissues, cancer environment can likely lead to their epigenetic dysregulation, possibly activating the expression of cancer-associated HERV proteins or transcripts forming double-stranded RNA (dsRNA) that could induce immune activation. HERV-encoded antigens expressed in a variety of cancers are reported to be targeted by cytotoxic T cells. In vitro, cytotoxic T cells that target HERV-K(HML6)-encoded epitope effectively lyse melanoma cells [62]. Patients with a past history of seminomas and limited healthy individuals also possessed defined HERV-K(HML2)-derived epitope recognized by CD8^+^ T cells [72]. In renal cell carcinoma (RCC) patients who received hematopoietic stem-cell transplantation, CD8^+^ T cells against an RCC-specific HERV-E antigen were produced after the transplantation and led to disease regression [73]. On the contrary, humoral response against HERV usually reflects disease severity of cancer. For instance, HERV-K(HML2)-reactive antibodies were found in the sera of germ cell tumor patients and the decreased titer correlated with longer survival [74]. Similarly, HERV-K(HML2) antibody reactivity in melanoma patients predicts poor prognosis [75]. Antibodies specific to cancer-associated HERV could therefore serve as an indicator of disease progression and treatment effectiveness.

In conjunction with the above, HERV elements are known to trigger innate immune responses due to their viral features (viral mimicry effect). HERV antigens (including nucleic acids or proteins) that are expressed in cells may act as pathogen-associated molecular patterns (PAMPs) that could be detected by the innate immunity pattern recognition receptors (PRRs), leading to the activation of innate immune responses, including downstream pro-inflammatory signals and the induction of subsequent adaptive immune responses [5,76]. Essentially, the host cell interprets the induced expression of HERV molecules as a sign of infection by an exogenous virus and mounts an antiviral innate immune response. For instance, it has been shown that the ERV avian leukosis virus-derived antisense long ncRNA in the chicken embryonic fibroblasts can stimulate the TLR3 pathway to induce an antiviral innate immune state against infection by other exogenous retroviruses [45]. Another example is the HERV-W Env, which can interact with receptors of the TLR4-lipopolysaccharide (LPS) pathway, including TLR4 and CD14, and induce pro-inflammatory cytokines such as IL-1β, IL-6, and TNF-α [77]. Finally, with implication for cancer treatment, it has been recently shown that radiation-induced ERV-associated dsRNA transcription in mouse melanoma cells promotes the activation of the innate antiviral MDA5/MAVS/TBK1 pathway, leading to the downstream transcription of IFN-stimulated genes and resulting in anti-tumor response [78]. The overall consequence of this viral mimicry is thought to weaken the cancer cells and attract cytotoxic T cells to the previously “immune-cold” tumor microenvironment, leading to tumor destruction.

In summary, HERV-encoded antigens/proteins could act as potential targets to trigger immune-mediated cascades that could be beneficial for tumor management. Provoking such mechanisms has promising significance in onco-immunotherapies.

## 11. Immunotherapeutic Strategies Targeting HERV for Cancer Treatment

Due to the potential contribution of HERV expression in certain diseases, it is theoretically feasible to target HERV expression via immunotherapies such as antibodies or vaccines. For example, studies have shown that some proteins encoded by individual HERV members are found in the active multiple sclerosis (MS) patients [79,80]. Temelimab, also known as GNbAC1, is a humanized IgG4 monoclonal antibody targeting the HERV-W Env protein that is associated with the pathogenesis of MS [81]. The therapeutic GNbAC1 antibody is designed to neutralize the impact of the HERV protein on inducing adverse pro-inflammatory responses associated with CNS inflammation and impaired remyelination. As such, neutralizing this HERV protein through GNbAC1 may elicit a desirable response against HERV-mediated pathophysiological mechanisms in MS disease progression. 

This HERV-targeting approach could similarly be applied for cancer treatment, and the development of prophylactic vaccines against HERV-derived antigens has recently gained attention as a plausible strategy, although the possible use of HERV as the target for anti-cancer strategies is currently limited by the still incomplete knowledge of their role in the development and progression of the various human cancers. It has been demonstrated that HERV-derived epitopes are recognized, processed, and presented as targets for T cell immune recognition [82,83,84]. Kraus et al. [85] developed a recombinant vaccinia virus that expressed HERV-K(HML2) Env protein (MVA-HKenv) and tested it in a syngeneic mouse tumor model where HERV-K Env was expressed in the murine renal cancer cells (RLZ-HKenv cells). Intravenous injection of RLZ-HKenv cells in the BALB/c mice led to metastatic lung cancer. A single vaccination with MVA-HKenv drastically resolved the pulmonary tumor nodules, suggesting the efficacy of the vaccine. The vaccinated mice displayed cell-mediated cytotoxic immunity against RLZ-HKenv cells, which can be explained by T-cell-mediated tumor cell lysis induced by the vaccine [85]. This group manifested an analogous study with HERV-K(HML2) Gag protein as the promising target to elicit a long-lived T cell response against tumor in the murine model [86]. A similar strategy has also been employed through adenovirus-mediated delivery of murine melanoma-associated retrovirus (MelARV) proteins Gag and Env to form virus-like particles displaying the cancer-associated MelARV Env to the immune system in mice, which could stimulate T cell response to inhibit murine colorectal tumor growth [87]. Likewise, another group investigated the extent of vaccination protection in rhesus macaques against ERVs as means to target HIV- and cancer-associated endogenous retrotransposable elements. Sacha et al. immunized this primate model with Gag and Env proteins of simian endogenous retrovirus (SERV), which resulted in the polyfunctional T cell expansion and humoral immune response against both SERV-encoded proteins [88]. In addition, there were no toxicity and adverse pathological findings related to vaccination, suggesting that vaccination against ERVs can safely induce immune responses and is a potential strategy for the management of HERV-associated neoplasms. Nonetheless, in spite of the fact that HERV-derived epitope-based vaccination has established a potential outcome in preclinical animal models, clinical evaluation for the safety of such a vaccination approach is required to encourage further immunotherapeutic progression targeting HERV in cancer therapy.

## 12. Inhibiting DNA Methylation to Induce Anti-Tumor Response through HERV Signature Upregulation as a Cancer Treatment Strategy

As mentioned earlier, HERVs are typically transcriptionally suppressed by DNA methylation in somatic cells [40], and the activation of HERV sequences through the inhibition of methyl transferases may contribute to viral mimicry effect and/or enhanced expression of neoantigens that can be detected by host immune surveillance [89]. The idea of inhibiting DNA methylation represents a potential strategy to tackle cancers associated with HERV signatures. Indeed, upregulated HERV expression has been observed following treatment with the DNA methyltransferase inhibitor (DNMTi) Guadecitabine (SGI-110) in hepatoma cells that are sensitive to its anti-tumor effect [90]. A similar observation was also found in ovarian cancer cells, in which a combination of the histone methyltransferase G9Ai and the DNMTi 5-aza-2’-deoxycytidine produced a synergistic anti-tumor effect that was associated with enhanced viral mimicry from the upregulated HERV expression [90]. Indeed, the use of DNMTi has been demonstrated to trigger TLR3 and MAVS-associated dsRNA sensing pathways that accompanied ERV upregulation in ovarian cancer cells, and this higher viral defense signature expression also correlates with tumor sensitivity to immune checkpoint therapy, particularly with the addition of DNMTi [91]. These observations therefore support the use of DNMTi for cancers associated with HERV signatures and provide impetus to further explore their use in onco-immunotherapies.

## 13. Antiviral Targeting of HERV as a Strategy for Cancer Treatment

Due to their similarity with HIV, it is theoretically possible to directly target HERVs through ARVs as an approach to defer their impact on disease progression. In the case of colorectal cancer, where HERV-H were found in almost 50% of samples [92], the transcription of HERV-H was strongly linked with early immune intrusion [65], microsatellite instability, lymph node invasion [93], and chemotherapeutic resistance [57]. The in vitro administration of cytotoxic chemotherapy in combination with antiviral therapy produced a significant anti-proliferative effect in tumors [57], suggesting the need to justify the clinical impact of HERV-H blockade in colorectal cancer treatment. This points to potential usage of antiviral therapy targeting HERVs as a treatment strategy to manage cancers. There are no specific antiviral drugs developed to target HERVs so far; however, several studies began to screen FDA-approved ARVs, which may effectively inhibit HERVs due to their close association with HIV. Although initially antivirals targeting viral RT were explored, ARVs that can block all significant steps of the retroviral life cycle could be considered. This is also supported by the observation that HIV patients may have a lower risk of developing MS than the others, possibly due to the suppression of HERV by ART [94].

Richa et al. tested both nucleotide RT inhibitors (NtRTI) and non-nucleotide RT inhibitors (NNtRTI) against HERV-K(HML2) RT enzyme activity in vitro. NtRTIs including abacavir, tenofovir, lamivudine, and stavudine showed significant and dose-dependent inhibition, whereas NNtRTIs including efavirenz, etravirine, and nevirapine also showed a significant inhibitory effect. Both drug groups had similar dose–response curves and IC_90_ values [95]. To determine the effect of RT inhibitors on HML2 infectivity, the same group tested VSV-G pseudotyped HML2 infection in HeLa cells in the presence of RT inhibitors. RT inhibitors such as abacavir and zidovudine both exhibited an inhibitory effect on HML2 infection in a dose-dependent fashion [95]. Contribution of RT activity in cellular transformation was also investigated in melanoma and prostate cancer cell lines. RT activity was halted by the use of NNtRTIs, such as nevirapine and efavirenz, and RT-encoding LINE-1 elements were downregulated by RNA interference (RNAi). Reduced RT activity resulted in downsized cell proliferation, enhanced morphological differentiation, and renovated gene expression patterns in vitro. Moreover, the tumorigenic phenotype of prostate carcinoma cells in the xenograft nude mice model was attenuated by the pretreatment with RT inhibitors [96]. It suggests that the endogenous RT may have a pivotal role in epigenetic regulation of cell proliferation and differentiation and may serve as a novel target in cancer therapy. Alongside, the inhibitory potential of HIV protease and integrase inhibitors against HML2 has also been investigated. Protease inhibitors such as darunavir and lopinavir readily docked to the protease catalytic site, thereby inhibiting HML2 protease activity, while the integrase inhibitor raltegravir inhibited the VSV-G pseudotyped HML2 replication in a dose-dependent manner [97]. This group also suggested that the replication of HML2 may be integration dependent, as the integrase inhibitor raltegravir showed a robust inhibitory effect against HML2 integrase [95]. Summarizing the above reports, it appears possible that the HERVs expressed in varied cancer types can be counter-attacked by employing ARVs used in HIV treatment.

## 14. Conclusions and Future Prospects

**Figure 1:**
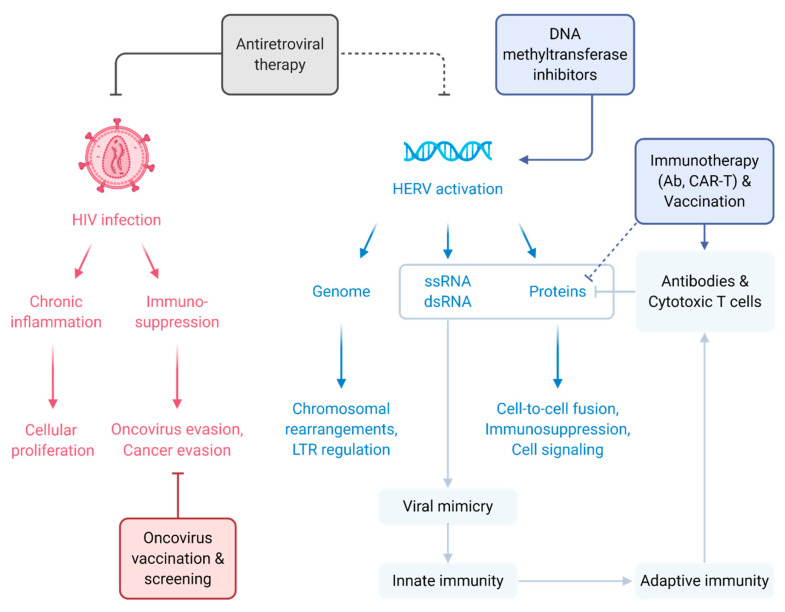
Illustration of the HIV– and HERV–cancer relationships and potential treatment strategies. HIV infection creates a dysregulated environment with chronic inflammation and immunosuppression that aid oncovirus transformation and cancer development (solid red arrows). Strategies to preclude HIV-related cancers include antiretroviral therapies (solid grey T bar) and oncovirus screening/vaccination (solid red T bar). As for HERV, activation and expression of HERV nucleic acid and protein elements have been found in various cancers, potentially contributing to cancer development (solid blue arrows). The aberrant expression of HERV elements has a viral mimicry effect that triggers innate and adaptive immune responses (solid light blue arrows), which have been exploited as approaches to fight HERV-associated cancers (solid light blue T bar). Inducing HERV expression through DNA methyltransferase inhibitors (solid dark blue arrow), immunotherapies targeting HERV proteins (dotted dark blue T bar and solid dark blue arrow), and antiretroviral therapies (dotted grey T bar) are being explored as potential anti-cancer therapies. Figure created with BioRender.com.

Like other animal retroviruses, HTLV and HIV have the potential (either directly or indirectly) to promote oncogenesis, leading to the increased incidence of a variety of human cancers in the infected population (Figure 1). In addition to this “traditional” scenario, HERVs are the age-old alien viruses residing in the human genome and exposing themselves in diverse human diseases. Increasing pieces of evidence indicate that the HERV gene expression is induced under specific physiological and pathological conditions. The exceptional depth at which cancer genomes are examined indicates that HERVs may have completely lost their direct tumor-causing ability due to insertional mutagenesis at the time of novel integration. Nevertheless, HERVs do still contribute to cancer pathology by distinct and often indirect mechanisms (Figure 1). Individual HERV members are selectively identified to possess tumor-associated properties that are significant in tumor carcinogenesis [50]. However, whether HERV is a causative agent or merely a consequence of the disease is still vague, since HERV activation or HERV-encoded protein expression at the disease microenvironment alone is insufficient to prove their pathogenic potency.

With a better understanding of the link between HIV infection and HERVs, it is evident that HIV can alter HERV gene expression in specific cell types. This means that, in addition to the well-characterized increased risk for HIV-associated malignancies, HERV dysregulation by HIV infection can further prompt such a vicious cycle, providing possible oncogenes and oncoproteins absent in physiological conditions. As described above, recent investigations have pinpointed that ARVs, beside reducing the risk for HIV-associated cancers, can also affect HERV gene transcription and HERV-encoded protein function. These data have been exploited to use ARVs as an anti-cancer modality in controlling HERV activation in a cancerous condition. Further findings described here suggest that HERV elements may also be restrained by HERV-epitope-specific vaccination and by monoclonal antibody therapy, suggesting the need for the development of immunotherapy for rapidly evolving cancer types. Due to HERVs’ background of retroviral origin and partial immune tolerance, these aliens inside us have become a potential target for future onco-immunotherapy. Understanding the role of HERV in cancer progression is analogously essential to building a steady approach to cancer management.
